# Two Cases of Genital Mal-Formation

**Published:** 1819-09

**Authors:** George Hume Wetherhead


FOR THE LONDON MEDICAL AND PHYSICAL JOURNAL.

*Two Cases of Genital Mal-formation.* Related by GEORGE HUME WETHERHEAD, M.D.

COMPLYING with your request, to put on paper a description of the cases of genital mal-formation, the particulars of which I casually mentioned to you, I commence with observing, that it formed part of my duty, when belonging to the flag-ship of Sir Richard Bickerton, at Spithead, to examine all seamen who were either drafted from the other ships of the fleet or impressed from the shore, to see that they were not disabled by rupture or otherwise. It was in the course of this duty that the two cases alluded to occurred.

The first was that of a seaman, sent on-board from H. M. S. Halifax. He was a slender-made young man, apparently about 18 or 19, but several years older by his own account; with small features, mild and feeble voice, fair complexion, and blue eyes: his neck was long and slender, his chest narrow and flat, and the mammæ were not at all protuberant; the upper and lower extremities were slim, and his fingers tapering. As he stood upright, his sexual developments seemed those of a woman: the mons veneris was very prominent, and distinct labia protruded from beneath; but, on closer inspection, a scrotum containing two small testes appeared, which the proximity of his thighs had concealed: this, too, was favoured by its situation being more retired than usual, seeing it was not borne forwards by a penis; in place of which, on separating the labia, a small clitoris, about half an inch in length, came into view, the glans of which had what seemed a meatus urinarius; but, on enquiry, I found that his urine did not pass by this orifice, but through three small apertures in a membrane occupying the site of the hymen, on each side of which the limbs of the scrotum (if I may so call them) commenced. I introduced a probe into the meatus of the clitoris, which proved a false passage, terminating under the skin behind the frænum. The apertures through which the urine flowed, were so placed as to resemble the position of the three angles of a triangle, and these so trifurcated the stream of urine, as obliged him to crouch down, as women do, when he passed it.

He affirmed that he had no sexual desires, and that the clitoris was incapable of erection. I got Dr. Denmark to see this example of deficient organization.

The second case was that of a tall robust young man, who had been just impressed, the peculiarity of whose conformation consisted in the absence of all vestiges of scrotum or testes. The penis was proportioned to his apparent manhood; but the skin descending thence was quite smooth and tense, and not a ruga of scrotum was to be perceived. The young man said that he was born so. No testis was to be felt in either groin; and he declared, what, from his athletic appearance, I was much inclined to doubt, that he had never experienced any amorous inclinations.

18, *Upper Montague-street, Montague-square;*
*29th July,* 1819.

